# State of the (t)art. Analytical approaches in the investigation of components and production traits of archaeological bread-like objects, applied to two finds from the Neolithic lakeshore settlement Parkhaus Opéra (Zürich, Switzerland)

**DOI:** 10.1371/journal.pone.0182401

**Published:** 2017-08-03

**Authors:** Andreas G. Heiss, Ferran Antolín, Niels Bleicher, Christian Harb, Stefanie Jacomet, Marlu Kühn, Elena Marinova, Hans-Peter Stika, Soultana Maria Valamoti

**Affiliations:** 1 Austrian Archaeological Institute (ÖAI), Austrian Academy of Sciences (ÖAW), Wien/Vienna, Austria; 2 Integrative Prehistory and Archaeological Science (IPAS/IPNA), University of Basel, Basel, Switzerland; 3 Office for Urbanism Zürich, Underwater Archaeology and Laboratory for Dendrochronology, Zürich, Switzerland; 4 Cantonal Archaeology of Zürich, Dübendorf, Switzerland; 5 Center for Archaeological Sciences, Katholieke Universiteit Leuven, Leuven, Belgium; 6 Royal Belgian Institute for Natural Sciences (RBINS), Brussels, Belgium; 7 University of Hohenheim, Institute of Botany (210), Stuttgart, Germany; 8 School of History and Archaeology, Aristotle University of Thessaloniki, Thessaloniki, Greece; University at Buffalo - The State University of New York, UNITED STATES

## Abstract

The site of Parkhaus Opéra is located on the north-eastern shore of Lake Zürich (Switzerland) and was documented during a rescue excavation in 2010 and 2011 by the Office for Urbanism, City of Zürich. Two charred bread-like objects were found in late Neolithic Layer 13 of the pile-dwelling, and are investigated using a novel set of analyses for cereal-based foodstuffs. Tissue remains of barley and wheat were identified, as well as a schizocarp of celery (cf. *Apium graveolens*), providing the first evidence for the use of bread condiments in the Neolithic. Cereal particle sizes were recorded and used to draw conclusions regarding milling and sieving of the raw material. Gas bubbles in the charred objects were measured in order to evaluate possible leavening of the dough. The outcomes of this research significantly advance the understanding of the production traits of cereal-based food during the Neolithic. The analytical techniques proposed by this study open up new possibilities for systematic and consistent investigations of cereal-based archaeological foodstuffs.

## Introduction

Is bread as old as cereal cultivation, or was it the outcome of a progressive development that culminated with the flourishing of the major civilisations of Classical Antiquity? Was prehistoric bread always the same culinary product that we know today, or that we can deduct from historical records? What was the role of bread in everyday alimentation? Has it always been as essential as it is regarded today in the western World? How should bread be defined after all, and where do we draw the line between bread and other cereal products created in the past?

As we know today, the scientific investigation of actual archaeological finds must always play a key role in the process of answering such questions [1–3]. It is such research which demonstrated the Upper Palaeolithic beginnings of (wild cereal) food processing as in Ohalo II [4–6], and the processing of cereals at Catalhöyük from the earliest Neolithic onwards [7]. The first “bread” finds from the Swiss Cortaillod culture [8] resulted in the scientific consensus that bread-making has been a part of central European food culture since the Neolithic. Most details in the history and development of bread-making are however still unclear in many aspects.

Regardless, no typology or standardised terminology exists for archaeological finds of cereal preparations. The habit of equalling archaeological finds to their presumptive modern counterparts has a long tradition. Widely used terminology such as Pumpernickel [9], cakes [10, 11], dumplings [11], or noodles [11–13] usually only consider the objects’ shapes and not the *chaînes opératoires* of their production. When referring to supposed “bread” finds in archaeology, very general definitions seem to be more appropriate. One definition may be that bread is a processed (and usually, but not always, cereal-based) foodstuff made of a variety of solid and liquid ingredients, optionally fermented, but eventually dried or cooked/baked [14, 15]. Such a broad definition of bread, if applied to present time, would encompass nearly all bakery and pastry products imaginable. There are also narrower definitions, some implying fermentation when using the term “bread”, opposing it to unfermented flat bread [16].

In light of this background, the aim of this study is to propose a standardised approach to record the parameters for archaeological “bread” finds. Morphological descriptions of prehistoric bread-like objects will be integrated with the microscopic analysis of their plant content. Moreover, procedures from material analysis will be applied to gain additional qualitative data on the components, as well as quantitative data on grinding and baking traits. The ultimate goal of this approach is the identification of (1) ingredients of the cereal products in order to link archaeobotanical crop spectra with actual food consumption, and also of (2) technological aspects of cereal processing in order to gain insight into the qualities of the products, the time invested in their production, and consequently their value and significance for a society [17–19].

As a case study illustrating this approach we selected two Late Neolithic finds of possible bread buns from the site of Parkhaus Opéra, Zürich, Switzerland, which are extraordinary due to their excellent preservation and precisely recorded find context. By using them as a link between archaeobotanical plant macroremains of cereals and actual culinary practices, we seek to expand the knowledge of local food culture in the lakeshore dwelling.

Additionally, we suggest that the development of an up-to-date classification scheme for archaeological finds of cereal products is a desirable goal for future research–in particular against the background of the artefactual character of bread [3]. For the time being, we suggest the use of more general terms for archaeological finds containing processed cereals. The current research combined with approaches followed within the scope of the ongoing EU Horizon 2020 project PLANTCULT (ERC-2015-CoG 682529) [20], will be used as the basis for a typology of bread remains.

### Taphonomy of cereal products

Most cereal products are the results of intentional removal or breaking down of the various types of plant tissues in the grains to varying degrees. The applied techniques include one or several processes in various sequences such as crushing/grinding/milling, sieving, soaking, boiling, fermenting, or baking/roasting, leading to improved palatability and digestibility (see short overviews in [14, 21, 22, 23]). However, these processes also greatly lower the resistance of these products to microbial decay as well as reducing their stability in water, which is why even under otherwise favourable waterlogged conditions, these objects will quickly disintegrate [3]. This explains why the preservation of waterlogged bread and other processed cereal objects is unknown until today. The only documented suspected waterlogged bread find from Ipwege, Lower Saxony [24], later turned out to be a bread-like idol consisting of mostly beeswax [25, 26].

Desiccated bread finds have indeed been recorded since the 19^th^ century [3], but are limited to extremely dry conditions in arid climates as documented by finds in Egypt [27] and Xinjiang, China [11, 28]. Cases of bread-like objects preserved by permafrost are unknown from the literature so far. Given the altogether unstable nature of cereal products in wet environments as mentioned above, for the time being we presume that archaeological cereal preparations (including breads) outside arid climates only preserve in a charred state.

Charred bread-like objects are not commonly found in prehistoric settlements. Among these, only lakeshore dwellings have resulted in relatively high numbers of intact finds of larger objects of cereal origin, including other equally rare and fragile remains such as complete cereal ears. As charred plant material is highly susceptible to mechanical stress [29], it is suggested that the deposition in water and gentle processes of sedimentation may play a major role as a selective force in the preservation of bread-like objects, not only by reducing pre-depositional fragmentation but also by minimizing post-excavational fragmentation by recovery techniques such as flotation [30].

There is also a seemingly high incidence of bread finds from ritual depositions in funerary contexts [16, 21, 31–35]. This may indeed emphasize a particular role that these bread-like objects may have played in burial rites [36], but may also just be the consequence of intentional (and careful) deposition, which therefore reduced the chance of shattering these fragile charred objects. Well-known *in situ* finds of intact breads in burned down Roman bakeries such as in Pompeii [37] or Carnuntum [38]–contexts where the objects did not undergo mechanical stress after charring–could also favour this hypothesis.

### Research history of archaeological bread-like objects

Some of the earliest attempts to identify the components of archaeological bread date back to the late nineteenth/early twentieth centuries, when the first lakeshore dwellings were excavated, as documented for sites such as Wetzikon-Robenhausen at Lake Pfäffikon [9, 39], or Station See at Lake Mondsee [40]. Despite the fact that histological knowledge at the time allowed for the identification of cereal bran under transmitted light (e.g. [41–43]), analysis of charred cereal products requires either a reflected light microscope with magnifications of 200-fold or more, or sometimes an SEM–both of which were not available at the time. Heer [9] leaves no doubt that several “breads” from Lake Pfäffikon must have been made of millet, without expanding upon the reasons for this conclusion. Hofmann [40], although favouring the same idea for the Lake Mondsee bread objects, at least concedes they might contain virtually any cereal given they had been thoroughly dehusked and finely ground. Although re-examination of such finds is still clearly needed, the “millet breads of Robenhausen” are still being cited uncritically (e. g. [36]).

Thanks to the pioneering work of Max Währen since the 1940s, archaeological finds of cereal products across Europe have attracted much attention, once again with a main focus on late Neolithic lakeshore settlements [44]. However, no analytical methods or criteria for Währen’s identifications and classifications are ever discussed in his oeuvre. Instead, a plethora of undefined terminologies renders the confirmation of Währen’s diagnoses difficult, if not impossible [45]. Re-evaluation of these finds with modern scientific methods is therefore among the main goals of bioarchaeological bread research, together with the establishment of a standardised typology of archaeological bread remains [14, 20].

Since the 1980s, research into archaeological bread remains has seen a revival beginning with the first archaeobotanical applications of cereal bran identification in archaeobotany [46–48]. Delwen Samuel’s work [3, 27, 49–52] must of course be mentioned in which she extensively explored the *chaîne opératoire* of Ancient Egyptian bread-making. Likewise, Ann-Marie Hansson’s contributions linking the archaeobotanical analysis of Viking Age cereal preparations [22, 31, 32, 34, 53] to historical and ethnographical accounts must be mentioned as a milestone in bread research. Since this period, a certain overlapping with the analysis of “food crusts”–charred food residues from inside pottery vessels–can be observed concerning general research questions on components and production traits [23, 54], as well as the applied methodologies [55, 56].

Contemporary analyses of the microstructure of charred food preparations include the application of two opposing procedures: (1) dissolution of the food remains: chemically dissolving incompletely charred parts of the amorphous aggregations, thus separating the entirely charred components and making them observable under the transmitted light microscope [55, 57, 58], or (2) observing intact microstructures *in situ*: leaving the amorphous matrix intact, identifying the components on preferably fresh fractured surfaces using a reflected light microscope or SEM [7, 21, 56, 58–60]. The latter approach can also serve as a source of information for the *chaîne opératoire* of bread-making e.g. by inferring flour grain sizes from measurements of cereal fragments [7, 21]. Identification of possibly fermented dough has usually been made either by qualitative descriptions of the gas bubbles found in broken faces [44] or by applying a threshold height on charred archaeological bread finds, separating them into raised bread (*pain*, higher than 25 mm) or unleavened flat bread (*galette*, lower than 25 mm) [16]. Quite recently, methods delivering quantitative data on pore sizes have been proposed and are currently under evaluation [21].

Chemical analyses have already delivered a few indications of some constituents in food preparations [16, 54], although often not in an unequivocally conclusive way. Furthermore, many archaeological “bread” finds which were unearthed prior to the 1980s and which require re-evaluation, are often not apt for chemical analysis due to sometimes excessive treatment with consolidating agents [61].

### The site of Parkhaus Opéra

**Fig 1:**
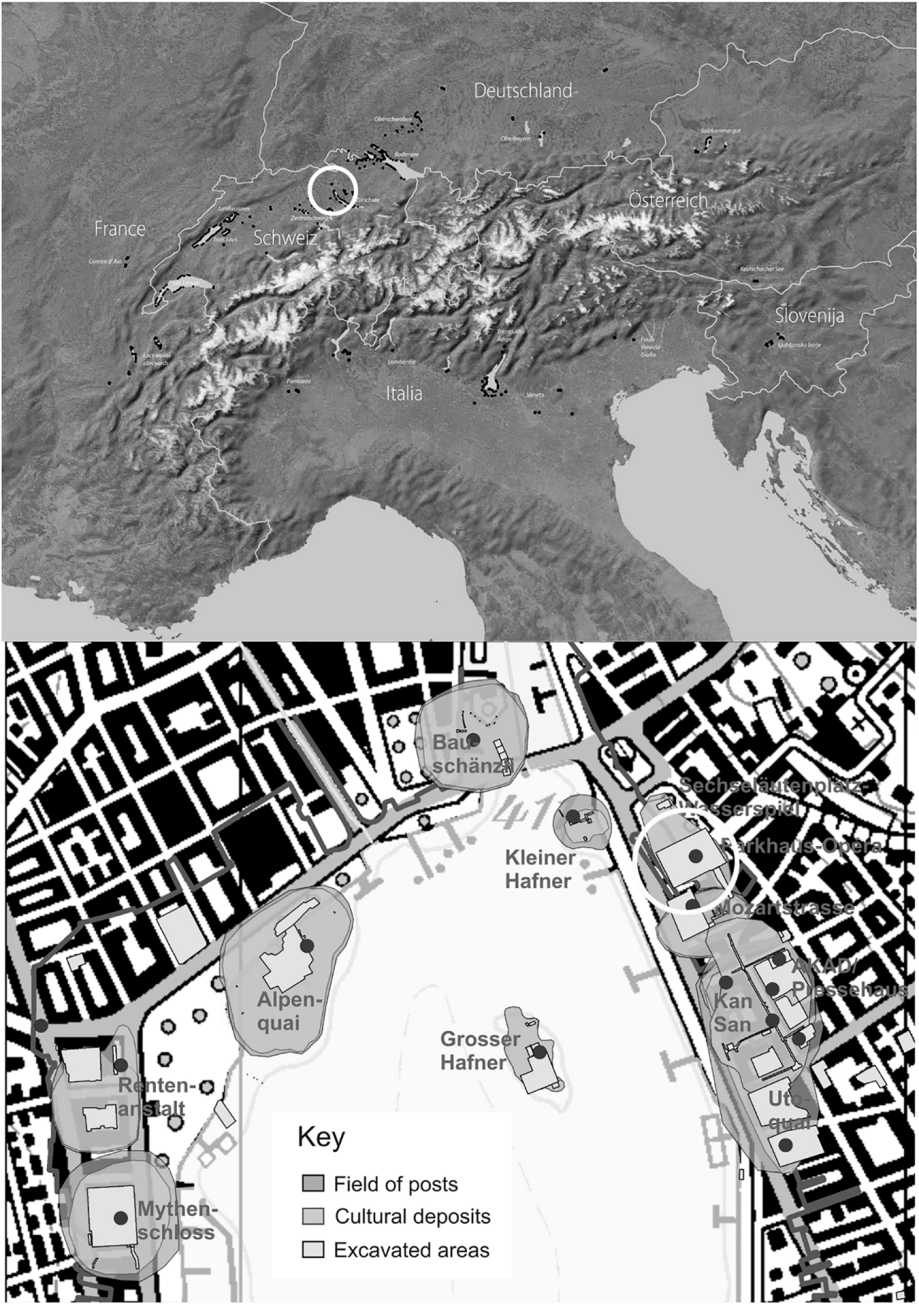
The site of Parkhaus Opéra. Location of Lake Zürich within the Alpine region (above) and plan of Parkhaus Opéra and other prehistoric pile dwellings around Lake Zürich (below). Image: Office for Urbanism Zürich / N. Bleicher.

**Fig 2:**
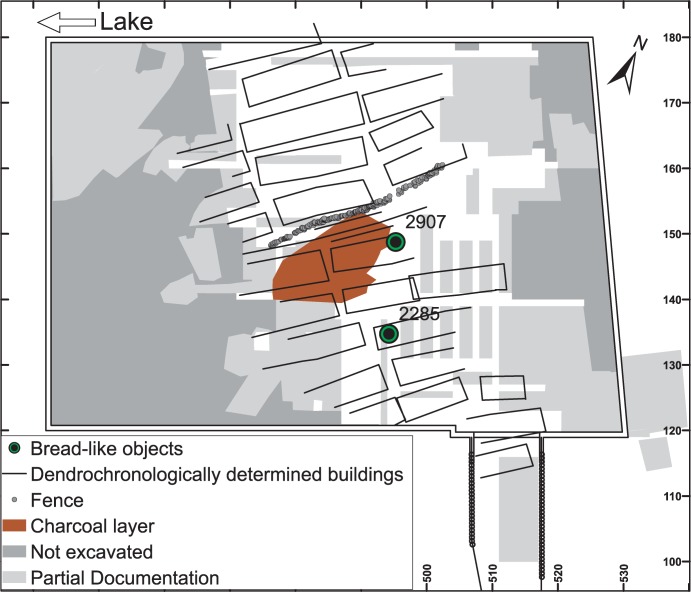
Find location. Section of the excavation at Parkhaus Opéra. Positions of find no. 2010.012.2285.3 = 2285 and find no. 2010.012.2907.5 = 2907 in Layer 13 are indicated in relation to buildings and the charcoal layer of a catastrophic fire event. Image: Office for Urbanism Zürich / N. Bleicher.

**Fig 3:**
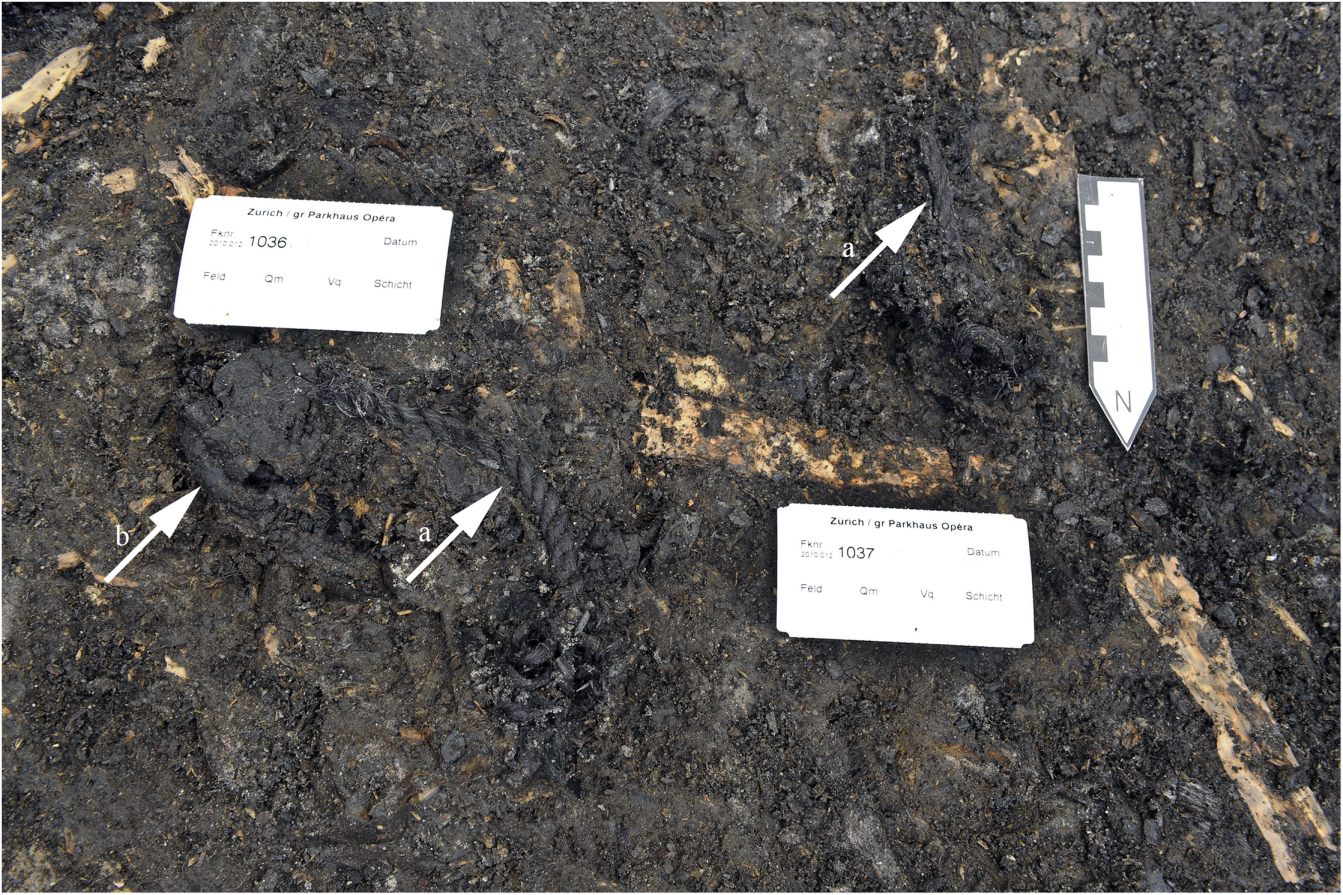
Layer 13 during excavation. Illustration of the state of preservation of organic matter in the cultural layer, in direct vicinity of find no. 2907. Wooden remains are visible as light-brown objects. Arrows indicate a) Rope fragments and a b) Bracket fungus fragment. Image: Office for Urbanism Zürich / N. Bleicher.

The site is situated in central Zürich close to the current shoreline of Lake Zürich (Fig 1). A large-scale rescue excavation in 2010 revealed six settlement layers and piles from eight settlement phases (Fig 2). The layers were in very different preservation states. While the oldest deposit contained nearly no organically preserved items, the two following layers showed extraordinary waterlogged preservation (Fig 3) with some leaf fragments still green. The younger three layers had experienced less favourable conditions and exhibited greater variability of conservation states and fewer organically preserved objects. Dendrochronological dating showed that the oldest deposit dates to the years between 3234 and 3226 BCE, while the youngest settlement phase dates between 2735 and 2727 BCE [62].

Layer 13, which was the best-preserved deposit, was documented over an area of more than 3000 m^2^ and spans a period of ca. 20–25 years between 3176 BCE and 3153 BCE. A total of 27 buildings as well as a fence/footbridge were identified and used for dendrochronological analyses [62].

### State of research on local plant-based subsistence and on cereal processing

Archaeobotanical analyses at the site allowed for the identification of more than 200,000 plant macroremains (mostly seeds and fruits) in about 250 systematically taken surface samples of 3–10 litres each, with a particular focus on large-seeded remains which are traditionally underrepresented in the small investigated volumes of sediment samples from sites with similar conditions of preservation [63], adding up to ca. 1000 litres of gently-sieved sediment using the “wash-over” technique [64]. Domesticates were well represented in the record, particularly cereals and oil plants. Among the cereals, emmer (*Triticum dicoccum*), and naked wheat (*Triticum aestivum/durum/turgidum*, mostly belonging to the *durum/turgidum* group) are better represented in the uncharred record, while barley (*Hordeum vulgare*, multi-rowed and mainly of the naked type) is one of the most important cereals when considering the remains preserved by charring. Oil plants including flax (*Linum usitatissimum*) and opium poppy (*Papaver somniferum*) were found in very large amounts, as is typical for other lakeshore sites [65–69]. Pulses were underrepresented in the samples due to difficulties during the identification process, currently resulting in the identification of only one find of pea (*Pisum sativum*). Large-seeded wild fruits such as hazelnuts (*Corylus avellana*), acorns (*Quercus* sp.) and wild apples/pears (*Malus sylvestris*/*Pyrus pyraster*) were also observed to play a significant role in the economy of the settlement [70, 71].

Besides the systematic bulk samples presented above, visible concentrations of well-preserved charred and uncharred materials were also collected. Among these finds were the charred objects discussed in this study which turned out to be cereal-based.

Plant food resources were obviously very important for the subsistence of the inhabitants of the site. The form in which they were consumed is not always clear, as the type of remains (mostly chaff) that end up embedded in the cultural layers are usually the refuse obtained from their culinary processing, and not the final products themselves.

Through the analysis of the spatial distribution of the largest concentrations of chaff remains of the different cereal taxa it was possible to determine that cereal processing took place in different forms for different cereals. Chaff of naked wheat and barley was found in accumulations both inside the limits of constructed features as well as in intervening spaces and along the central palisade. Quite differently, emmer chaff was mostly found in rubbish heaps that accumulated under the floors of those buildings. These cereals were therefore most likely grown, stored, and potentially also consumed separately.

Was there any evidence of further culinary processing of these grains? Besides chaff remains, fragments of bran can also preserve in waterlogged contexts [48, 72–75]. These were not identified at the species level due to the expenditure of time required, while their identification as cereals is straightforward. Fragments of bran were indeed found all over the excavated area, but in some larger concentrations in buildings located to the south of the settlement.

Given the spatial relation of grinding stones and cereal bran fragments in the southern half of the village, it was suggested that these fragments could have originated during cereal grinding (although other routes of entry such as faeces cannot be excluded). The current interpretation is that cereal grinding may have been frequent at least in this southern part of the site and that cereals were consumed in a transformed state, in smaller particles [76]. This is precisely the area where the charred objects presented in this paper were found.

## Material and methods

### Terminology

As stated in the introduction, finds like the ones analysed in the current study have often been classified using descriptive terms lacking clear delimitations. The authors therefore propose to avoid the use of any direct analogies to modern cereal products (“doughnut”, “porridge”, “cake”, “biscuit”, etc…) until a standardised and stringent typology for archaeological bread finds is established. Instead, we propose the general terms “cereal preparation” or “cereal product” for any amorphous charred object (AOV–amorphe Objekte verkohlt; OAC–objets amorphes carbonisés; MOC–matière amorphe carbonisée, BGF–Brot, Gebäck, Fruchtfleisch) [21, 77–80] with positive evidence for cereal content. For cereal preparations with a recognizable outer shape, we propose the general term “bread-like object” as used throughout this paper.

### Study permissions and sample repositories

The institutions eligible to issue study permits–the Office for Urbanism Zürich and the Cantonal Archaeology of Zürich–were the contracting entities of the current study, and both are represented by co-authors. Therefore, no additional permits were required for the described analyses.

Specimens are accessible to scientists on demand in the permanent repository of the Cantonal Archaeology of Zürich / Kantonsarchäologie Zürich, Stettbachstrasse 7, 8600 Dübendorf, Switzerland. Contact: are.archaeologie@bd.zh.ch / +41 (43) 259 69 00. The SEM subsamples are accessible to scientists on demand in the permanent repository of the Austrian Archaeological Institute, Head Office Vienna, Franz Klein-Gasse 1, 1190 Wien, Austria. Contact: mailbox@oeai.at / +43 (1) 42 77–271 01

### The bread-like objects

Two finds were examined in this study. For further ease of use, their find numbers will be shortened, and referred to in abbreviated form (find no. 2010.012.2285.3 = 2285, and find no. 2010.012.2907.5 = 2907). In Layer 13, the two finds lay within the plant detritus, forming the matrix of the deposit (Fig 3). Find no. 2907 was found near a layer of charcoal, find no. 2285 several metres away (Fig 2). There is no indication that the items were charred during the catastrophic fire which was documented for a sector of Layer 13 [81].

**Fig 4:**
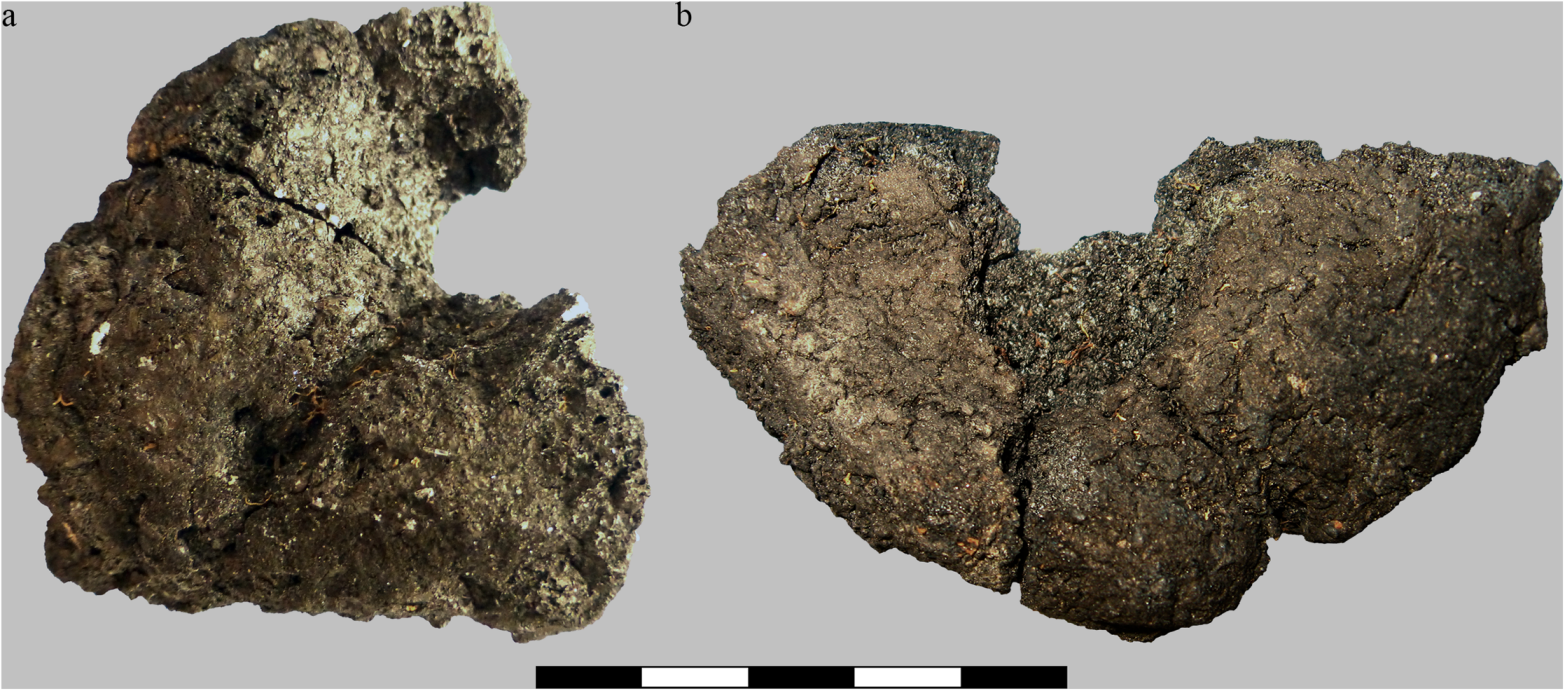
Overview of the two analysed bread-like objects. Possible upper surface of a) Find no. 2285 and b) Find no. 2907. Centimetre scale. Image: ÖAI-ÖAW, VIAS / A. G. Heiss.

Upon excavation, find no. 2285 was recovered broken into two fragments (Fig 4). When fitted together, the resulting shape is that of approximately one half of an irregularly disc-shaped object, with a total diameter of ca. 6 centimetres. We suggest that this was probably also the original diameter of the entire charred object. The low ratio of its maximum diameter vs. maximum height (2.8: 1, see Table 1) gives the find a rather bulky appearance. The inner walls of the find’s eccentric hole resemble the outer surfaces of the rest of the object, which is why it seems likely that the hole is indeed an original feature of this bread-like object.

**Table 1:** Basic description of the two bread-like objects from the excavation at Parkhaus Opéra.

	Find no. 2285	Find no. 2907
Total weight [g]	10,0	12,1
Total max. diameter [mm]	59	101
Total max. height [mm]	21	19
Radio max. diameter: max. height	1.3: 1	5.3: 1
Number of fragments	2	3
Max. fragment dimensions [mm]	59 x 35 x 21	65 x 38 x 19
L x W x H	37 x 27 x 16	47 x 36 x 16
		15 x 15 x 9

Find no. 2907 was also recovered in fragmented state (Fig 4). Only two larger fragments could be unequivocally associated to each other (i. e. fitted together), while the third and smallest one could not be fitted, and was not considered in the current analyses. Similar to find no. 2285, find no. 2907 also represents roughly one half of a disc-shaped object. While its diameter measures 10.1 centimetres and therefore nearly twice as much as of the aforementioned find, its maximum height is indeed lower (Table 1). Just like find no. 2285, find no. 2907 also bears a more or less eccentric hole. However, its jagged outline in combination with the appearance of the surface within the hole, which differs greatly from the surface of the undamaged area of the object, strongly suggest that this hole is not an original feature of the find, and must have originated from mechanical stress after charring.

### Sample preparation

The supposedly post-depositional fractured faces of both bread-like objects were examined without pretreatment under a light microscope (Olympus SZH10 binocular) with magnification up to 70x. Images were acquired using a Canon Powershot A540 camera and an ocular adapter hand-tailored by R. Mehnert (Weil der Stadt, Germany). The limitations in depth of field were compensated for by stacking multiple images with the software Helicon Focus [82].

No fresh fractures were produced in the objects to prevent any further damage, with the exception of the SEM samples. These, each measuring ca. 2 cm³, were taken from the existing fractured faces. Both subsamples were sputter-coated with gold/palladium, and analysed using a Zeiss EVO 60 XVP [83, 84].

### Component analysis

**Fig 5:**
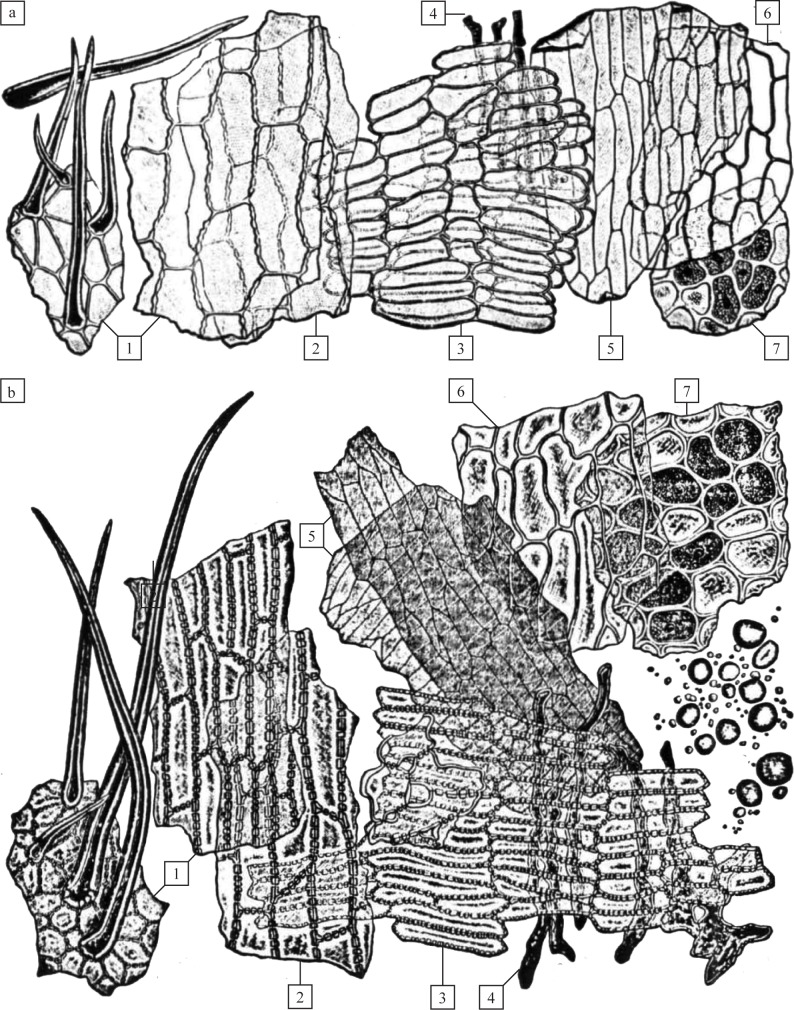
Diagnostic cereal tissue types as visible in plane sections. Species: a) Barley (*Hordeum vulgare*), and b) Bread wheat (*Triticum aestivum*). Tissue types: 1) epidermis at the apex (left) and in the middle of the grain (right), 2) Hypodermis, 3) Cross cells, 4) Tubular cells, 5) Testa, 6) Perisperm, 7) Aleurone. Applied diagnostic features: 3a) Thin-walled cross cell walls in barley, often in a double layer (the latter not visible under SEM), and 3b) Thick-walled, regularly and conspicuously pitted cross cells in wheat species ([41], modified).

**Fig 6:**
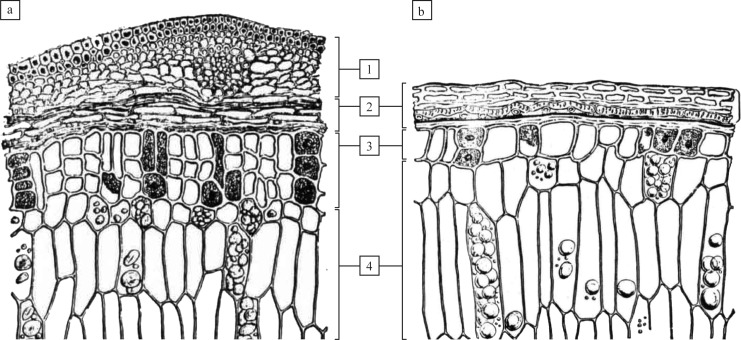
Diagnostic cereal tissue types as visible in cross sections. Species: a) Barley (*Hordeum vulgare*), and b) Bread wheat (*Triticum aestivum*). Grain parts and tissue types: 1) glume, 2) Pericarp and seed layers (corresponding to Fig 5, numbers 1 to 6), 3) aleurone tissue, 4) endosperm. Applied diagnostic features: 3a) Double to triple layer of aleurone in *Hordeum*, in contrast to 3b) Typically single layers as in *Triticum* and all other Old World cereal crops ([41], modified).

Histological identification of cereal tissues was carried out following Heiss et al. [21], using as identification criteria the anatomical structure of the cross cells’ (= transverse cells) walls (Fig 5), the arrangement of aleurone cell layers (Fig 6) and glume epidermal features [41, 85–88]. Identification based on the features of cross cells was limited to genus level, as e. g. differentiation of *Triticum* species is only possible if the original tissue location on the grain is known [47]. We did not follow the approach of using cross cell length as a means of distinguishing the genera *Triticum* and *Hordeum* [7] for a similar reason: cross cell length is too variable within the surface of a single grain to bear significance in identification [47, 48]. The millets *Panicum miliaceum* and *Setaria italica*, which can easily be identified by their glumes if present [43, 89], cannot be satisfactorily differentiated from barley by their cross cells alone [41]. As the significance of millets as food crops in Neolithic Europe can certainly be ruled out due to their absence in the period considered [90, 91], cross cells meeting the diagnostic criteria in Fig 5 are considered as originating from barley in the current paper.

Identification of seed admixtures was carried out using the ÖAI/ÖAW Bioarchaeology Department’s reference collection, as well as standard identification literature [92–96]. Experimental charring of *Apium graveolens* was carried out in a Nabertherm muffle furnace, simplifying an existing charring protocol under low oxygen / anoxic conditions [97]: temperature was set to 270°C for 6 hours, and the material (origin: University of Hohenheim [UHOH], IS 1990, Nr. 1413) was embedded in purified sea sand in order to establish a low-oxygen atmosphere.

### Measurements

For the evaluation of overall cereal fragment sizes, maximum dimensions of each fragment visible in the fractured faces of the objects were manually recorded using the software ImageJ [98]. These measurements were carried out on (supposedly post-depositional) fractured faces of both bread-like objects, recording all fragments visible with 10-fold magnification under a light microscope, as well as those visible with 100-fold magnification under the SEM. The results, presented in histograms (100 μm steps), are grouped according to modern equivalents of ground cereals [99, 100]: flour and dunst (particles smaller than 300 μm) vs. semolina (300–1000 μm) vs. grist (larger than 1000 μm).

The modern baking industry has applied a broad range of analytical methods in routine quality control for decades. While many of them can only be applied to unbaked (and uncharred) dough, such as the observation of rheological parameters, gas retention, or gluten composition [101–103], some others seem worth exploring for archaeobotanical charred material. The structure of baked bread, a matter technically being a stable foam, can be described by parameters such as pore shape, pore density and connectivity. In the modern bread industry, these factors are measured to assess baking conditions [104, 105].

For the charred bread-like objects presented in the current study, we use a simplified protocol for measuring pore sizes initially developed for pottery [106], which was later adapted for modern bread [104] and eventually for archaeological bread finds [21]. We then compare the results to the older dichotomous classification of archaeological bread finds into raised bread (*pain*, higher than 25 mm) and unleavened flat bread (*galette*, lower than 25 mm) [16].

**Fig 7:**
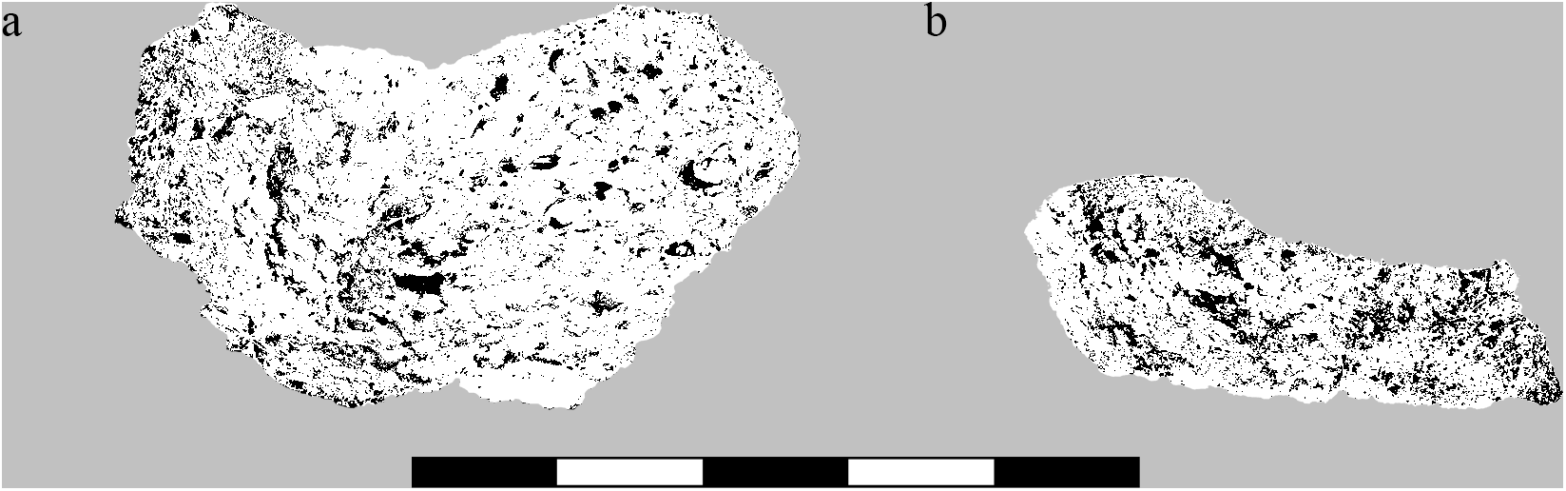
Cavity measurement. B/W images used as raw data for image analysis in a) Find no. 2285, and b) Find no. 2907. Centimetre scale. Image: ÖAW-ÖAI, VIAS / A. G. Heiss.

For the current material, the broken faces of both objects were photographed under skewed light, resulting in a highly contrasted image. After generating a black and white threshold image (Fig 7), ImageJ’s [98] “fit ellipse” function was used to automatically count all black areas (cavities) in the observed areas. Measurements below 100 μm were ignored in order to avoid artefacts caused by single pixels. Only the resulting maximum diameters of cavities were evaluated.

## Results

### Components

#### Find no. 2285

**Fig 8:**
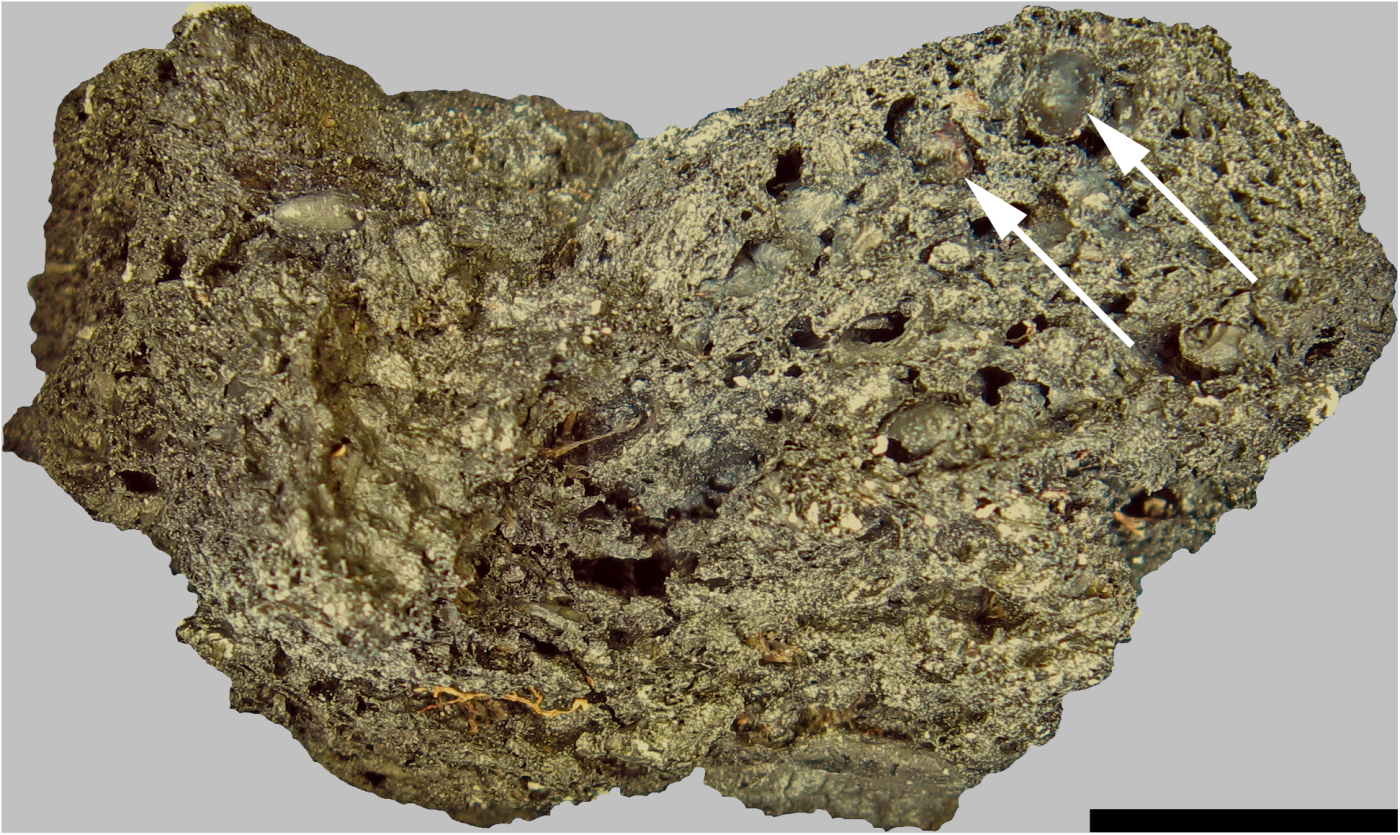
Overview of a fractured face of find no. 2285. Arrows indicate grains identifiable as barley (*Hordeum vulgare*). Scale bar length: 1 cm. Image: ÖAW-ÖAI, VIAS / A. G. Heiss.

**Fig 9:**
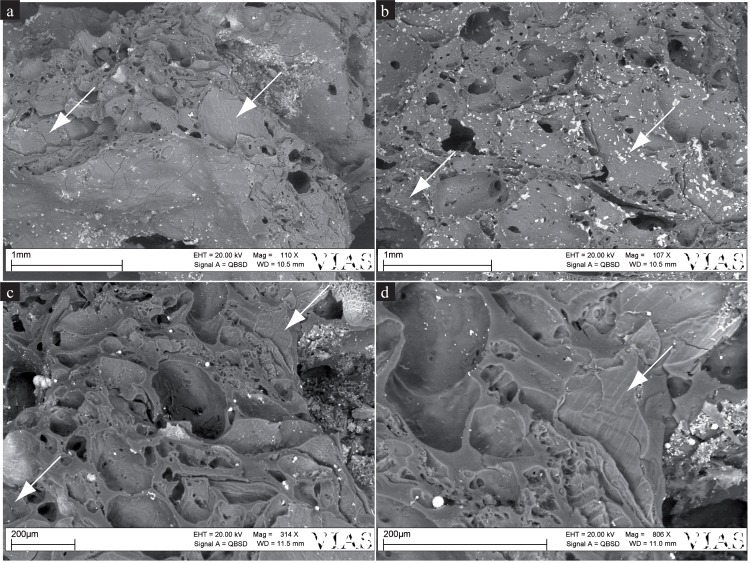
SEM images from find no. 2285. Arrows indicate cross cell patches. a-c) Overview images, d) Detailed view of c, showing thin-walled cross cells as found in barley (*Hordeum vulgare*).

Of the larger grain fragments visible in the fractured faces, two could be identified as barley (*Hordeum vulgare*) due to their grain outline (see Fig 8). Under the SEM, no cross sections of aleurone tissue allowing for verification of barley as a component (vs. identification of other cereals) were visible, however numerous patches of cross cell tissue were found (Fig 9). No evidence of additional cereal taxa or of other admixtures could be recognised in the SEM sample. Starch grains were not observed.

#### Find no. 2907

**Fig 10:**
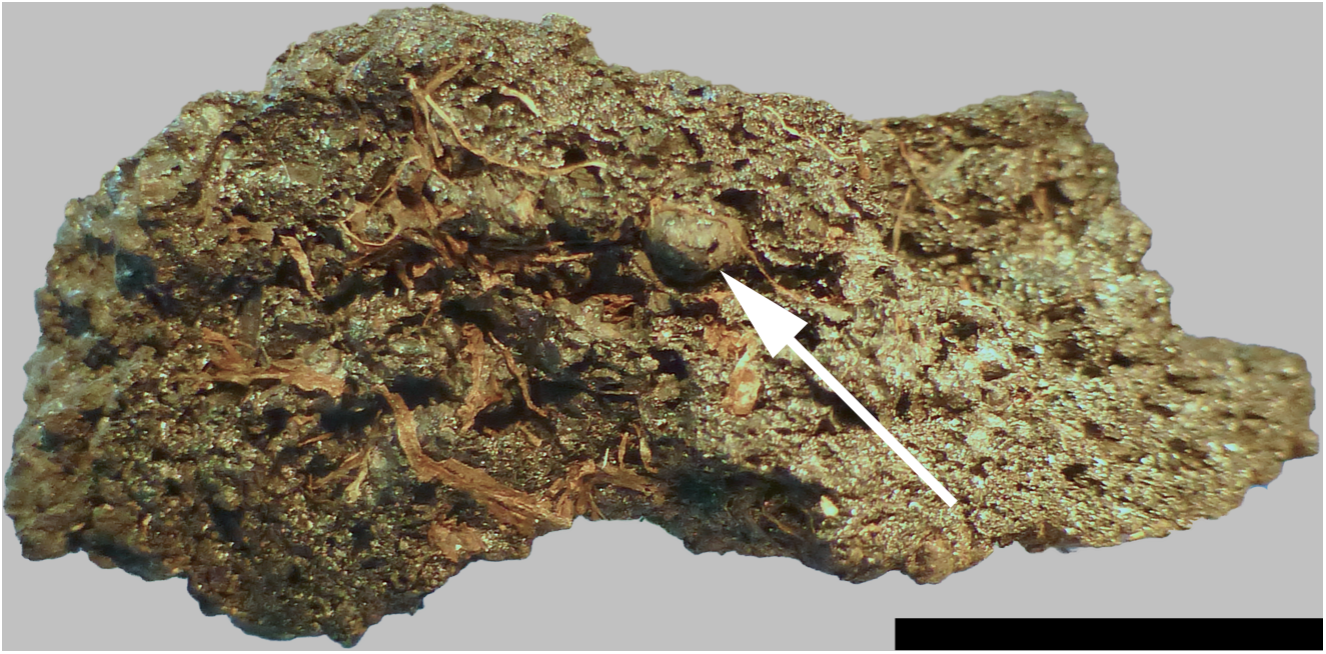
Overview of a fractured face of find no. 2907. The arrow indicates a grain identifiable as most likely barley (cf. *Hordeum vulgare*). Scale bar length: 1 cm. Image: ÖAW-ÖAI, VIAS / A. G. Heiss.

**Fig 11:**
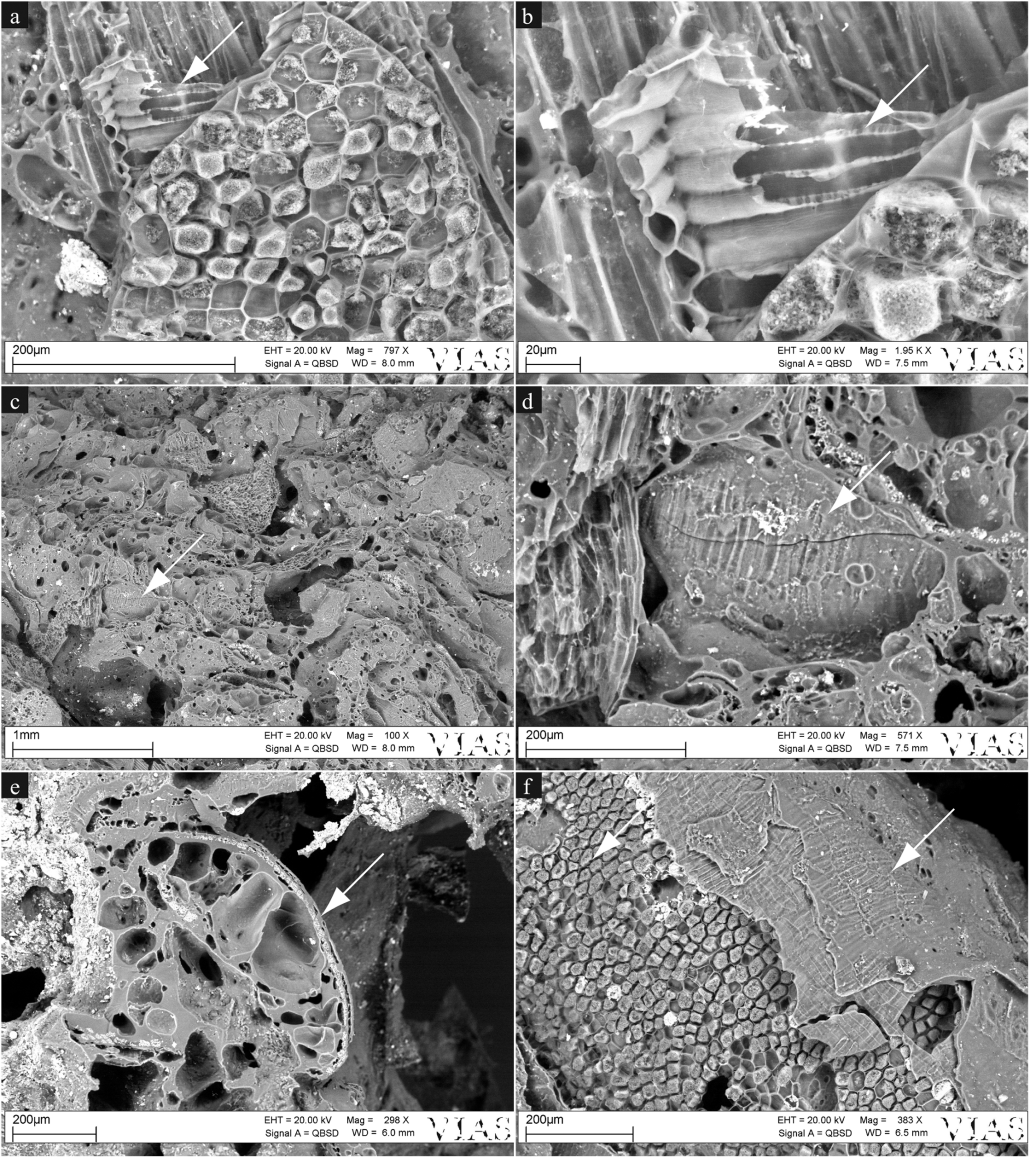
SEM images from find no. 2907. Arrows indicate regions of interest. a) Cross cells patch, covered by a patch of aleurone cells. b) Magnification of a, showing thick-walled and pitted cross cells as found in wheat (*Triticum* sp.). c) Cross cells embedded in the matrix. d) Magnification of c, showing the thick-walled and pitted cross cells of the *Triticum* sp. type. e) Portion of a cereal grain showing single-layered aleurone. f) Cereal grain showing several pericarp layers, from aleurone cells on the left to cross cells on the right hand side. Image: ÖAW-ÖAI, VIAS / A. G. Heiss.

Under low magnification (10x), one of the larger grain fragments was found to most likely derive from barley, although a definite identification was not possible (cf. *Hordeum vulgare*, Fig 10). Under the SEM, only hints of *Triticum* sp. (cross cells with thick, pitted walls) and of cereals other than barley (single-layered aleurone tissue) were observed (Fig 11). Starch grains were not observed in the sample.

**Fig 12:**
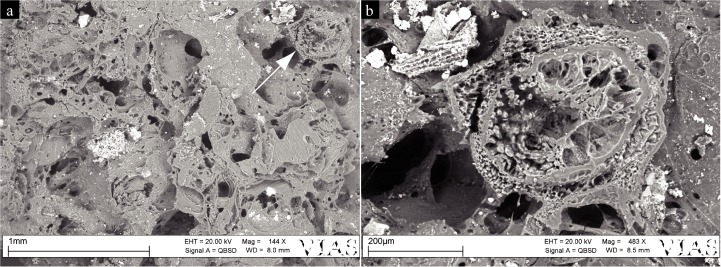
SEM image of the celery (cf. *Apium graveolens*) fruit. a) Embedded in the matrix of find no. 2907, b) In magnified view. Image: ÖAW-ÖAI, VIAS / A. G. Heiss.

**Fig 13:**
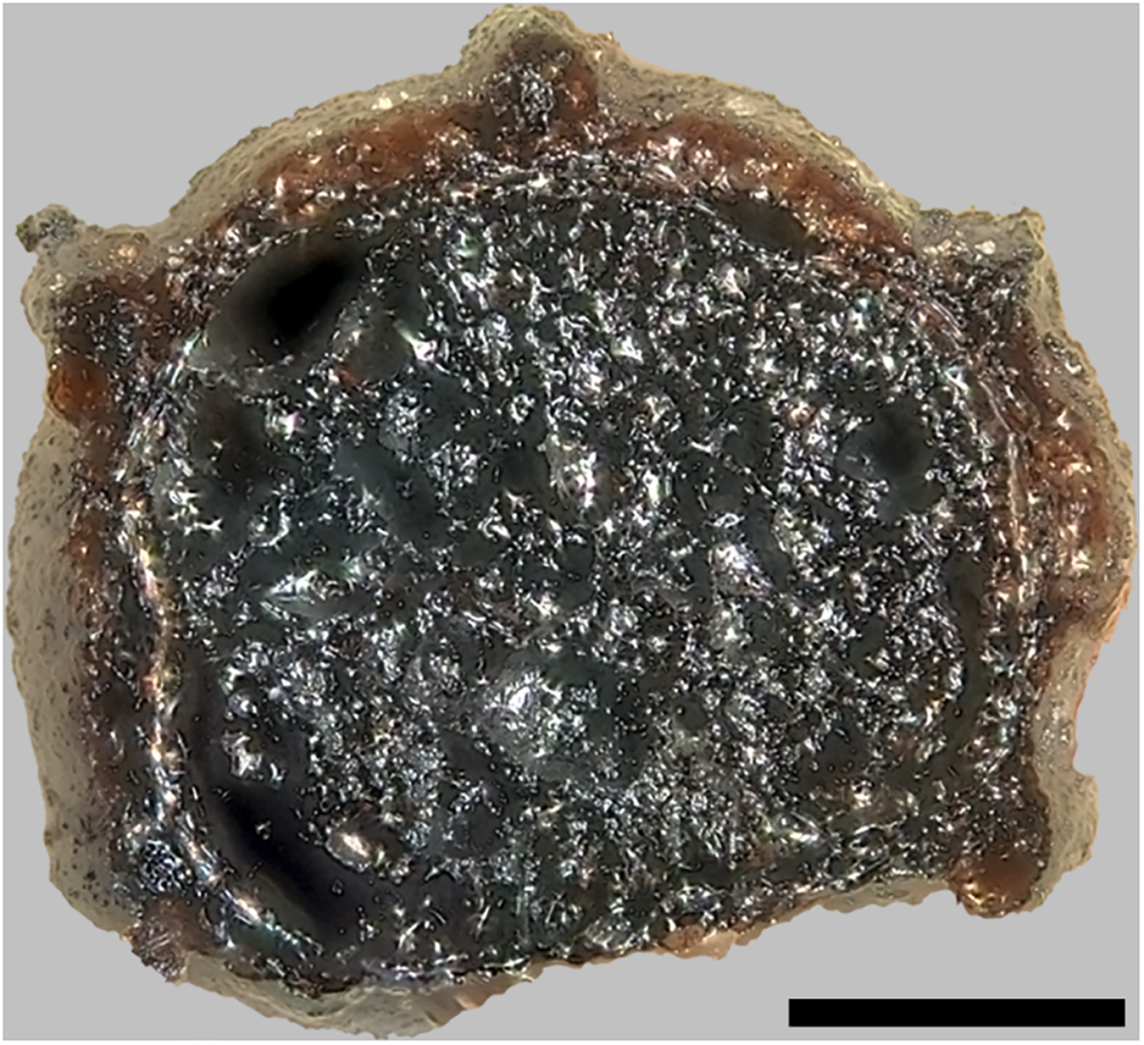
Experimentally charred schizocarp of celery (*Apium graveolens*). Scale bar length: 100 μm. Image: ÖAW-ÖAI / A. G. Heiss.

One sector of the SEM sample of find no. 2907 revealed a relatively isodiametric (ca. 0.5 mm) structure bearing three clearly visible dorsal ridges and two smaller lateral/ventral ones (Fig 12). A distinct differentiation between an outer hull and an inner portion is visible, both lacking clear cellular features, most likely due to charring. In spite of the limited available morphological features, the structure can clearly be identified as an Apiaceae schizocarp. Comparison of size and shape with European Apiaceae reference material as well as with experimentally charred material (Fig 13) resulted in the identification as very probably celery (cf. *Apium graveolens*).

### Structure

#### Find no. 2285

The overall macroscopic and microscopic structure of the object was very heterogeneous, with irregular distribution of larger cereal grain fragments and larger cavities. No clear differentiation of an inner crumb and an outer crust was observed.

**Fig 14:**
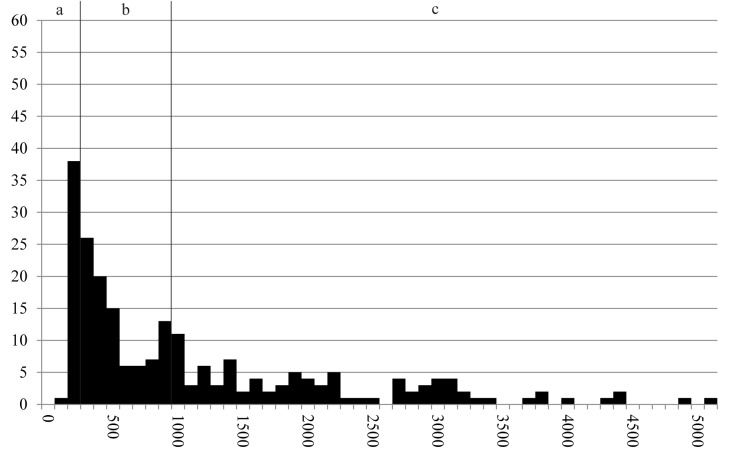
Histogram of the recorded maximum dimensions of components (N = 223) in find no. 2285. Y axis: number of occurrences, Y axis: size class in micrometres (in 100 μm steps). Grouping is according to grain sizes of modern grain products: a) Flour and dunst (smaller than 300 μm), b) Semolina (300–1000 μm), and c) Grist (larger than 1000 μm). Raw data is given in S1 Table.

Fragment sizes of cereal grains were recorded for 61 grain fragments visible under the binocular, and for 162 grain/bran fragments via SEM imagery. The overall data (Fig 14) show a heterogeneous mixture, with fragment sizes ranging from roughly 0.1 to 5.6 millimetres. The entirety of measurements divides roughly equally into three groups: measurement ranges of modern dunst and flour (29% of measurements), semolina (300–1000 μm: 35% of measurements), and grist (36% of measurements).

**Fig 15:**
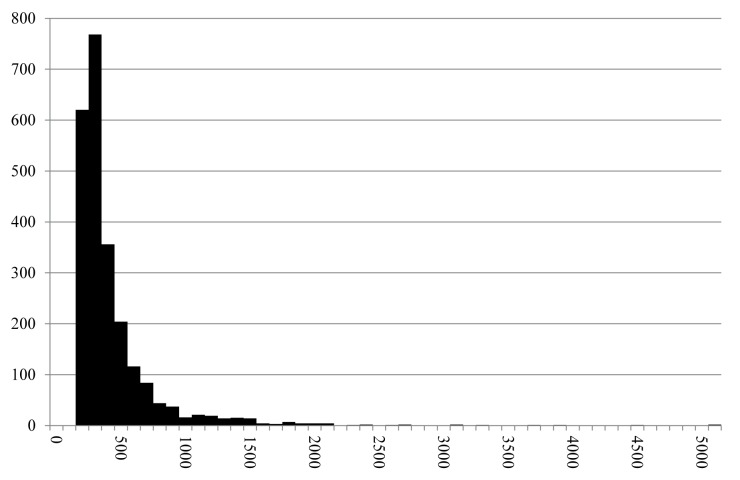
Histogram of the measured cavities (N = 2,368) in find no. 2907. Y axis: number of occurrences, Y axis: size class in micrometres (in 100 μm steps). Raw data is given in S2 Table.

Image analysis of cavities resulted in a total of 2,368 values ranging from 121 to 7013 μm, 95% of them are below 1 mm (Fig 15).

#### Find no. 2907

As in find no. 2285, the overall structure of the object could be described as very heterogeneous, but in general denser than the aforementioned find, large cavities were lacking. No clear differentiation between crumb and crust was observed.

**Fig 16:**
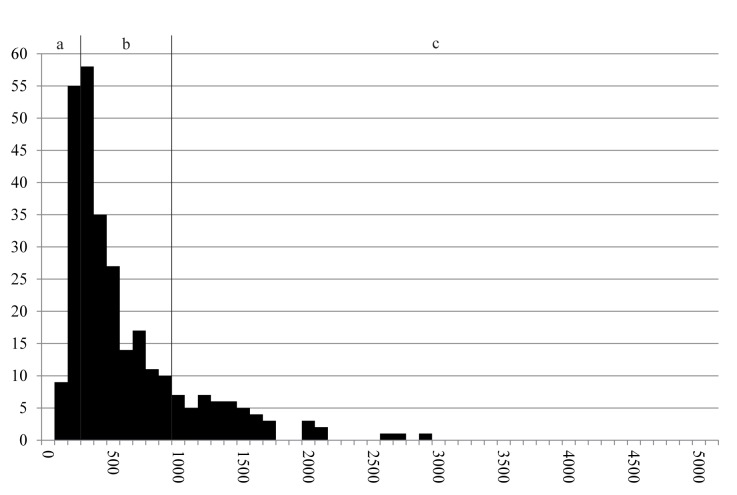
Histogram of the recorded maximum dimensions of components (N = 287) in find no. 2907. Y axis: number of occurrences, Y axis: size class in micrometres (in 100 μm steps). Grouping according to grain sizes of modern grain products: a) Flour and dunst (smaller than 300 μm), b) Semolina (300–1000 μm), and c) Grist (larger than 1000 μm). For raw data see S3 Table.

80 cereal fragments could be measured under the stereomicroscope, while the dimensions of 207 fragments were recorded using SEM. In spite of the minimum and maximum measures spanning roughly the same range as in find no. 2285 (0.1 to 5.7 millimetres), grain size distribution is strongly shifted towards smaller values (Fig 16): 43% of the measurements correspond to modern equivalents of dunst and flour, while 24% correspond to semolina and 15% to grist.

**Fig 17:**
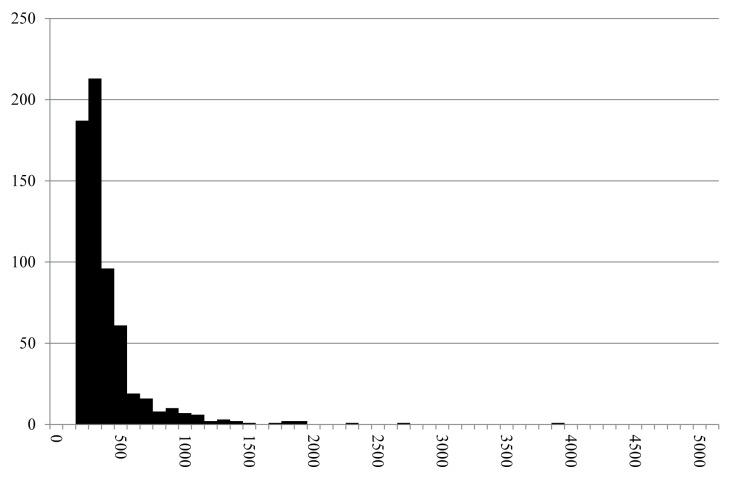
Histogram of the measured cavities (N = 639) in find no. 2907. Y axis: number of occurrences, Y axis: size class in micrometres (in 100 μm steps). Raw data is given in S4 Table.

639 cavities were measured by image analysis, spanning from 126 to 3894 μm. 97% of the values were smaller than 1 mm (Fig 17).

## Discussion

### Plant-based components

#### Cereals

In find no. 2285, all evidence indicatres *Hordeum vulgare* as main cereal ingredient, and no traces of other cereals were observed.

In find no. 2907, evidence for barley is uncertain (cf. *Hordeum vulgare*), while a wheat species (*Triticum* sp.) was also identified, therefore indicating a product made from mixed cereal meal. As inferred from the archaeobotanical find assemblage, one or several of *Triticum monococcum*, *T*. *dicoccum*, *T*. *aestivum/durum/turgidum*, or also *T*. *timopheevi* (*Triticum* sp. “new type”) are possible sources of the wheat material [76]. It should be noted that based on these results, past generalised implications such as “flat bread = barley bread” [107] should be regarded with even more caution.

#### Condiments

For the extraordinary find of a celery (cf. *Apium graveolens*) schizocarp in the matrix of find no. 2907, currently no comparable data are available from previous studies. This plant occurs in the archaeobotanical find assemblages of several pre-Alpine lakeshore dwellings despite not being native to central Europe [108, 109]. For Parkhaus Opéra it has not yet been documented [76], but is present nearby in more or less contemporary sites along Lake Zürich, such as at Zürich Kanalisationssanierung Seefeld phase 4, and at Zürich Mozartstrasse phase 3 [65, 110]. Previous works have suggested a link of imported Mediterranean condiments *Apium graveolens* and *Anethum graveolens*–the latter also directly documented for Parkhaus Opéra–and salt trade with northern Italy [111]. Whether this may also indicate the possibility of salt as an ingredient of find no. 2907 or not, it would stand to reason to interpret this find of celery as the first evidence for a bread condiment of the central European Neolithic.

#### Quantification

A question of both archaeological and public interest concerns the quantification of ingredients, as it often occurs that the public eventually expect “recipes” for certain ancient foodstuffs after scientific analyses.

Considering this, one must keep in mind that the acquired quantity of taxon-specific data for both analysed objects is very limited with the applied methods. Very few surfaces can be analysed under SEM and used as a data source for taxon recognition, while the majority of the object remains unobservable. Therefore it must be emphasised–even more so than for the interpretation of “normal” archaeobotanical assemblages–that the lack of evidence for a certain taxon in a ground cereal product can by no means be interpreted as a proof of its absence. The same limited explanatory power applies for quantities, clearly not allowing for an interpretation of relative quantities between cereals in compound preparations such as in find no. 2907.

Quantitative assessments of admixtures, be they intentional (condiments) or accidental (weeds, chaff), seems to be even less sensible due to their expectedly low proportions in cereal products in comparison to the main ingredients i.e. the cereal grains/flour due to the fact that: (1) accidental admixtures can be expected to be strongly reduced by the known *chaînes opératoires* of cereal processing and bread-making [14, 22, 45, 112–116], and (2) intentional admixtures such as condiments can only be expected in minor amounts, or at least this is what historical recipes from the Roman period [117, 118] suggest.

As a summary, the general probability of discovering any condiments in the few observable surfaces of a charred cereal preparation is minute, rendering the celery (cf. *Apium graveolens*) schizocarp in find no. 2907 an exception, and one that will give no hints to the quantities used in the dough preparation.

### Structure and production traits

#### Grinding

Grain size measurements of the two analysed finds results in two very different grain size distributions: While both objects definitely contain finely ground cereal meal mostly comparable to modern dunst and semolina, find no. 2285 displays an overall high degree of heterogeneity with a strong tendency towards larger fragments. This can be interpreted as either an intentional mixture of flour/dunst with semolina and grist, or as the result of quickly and incompletely grinding the grains.

**Fig 18:**
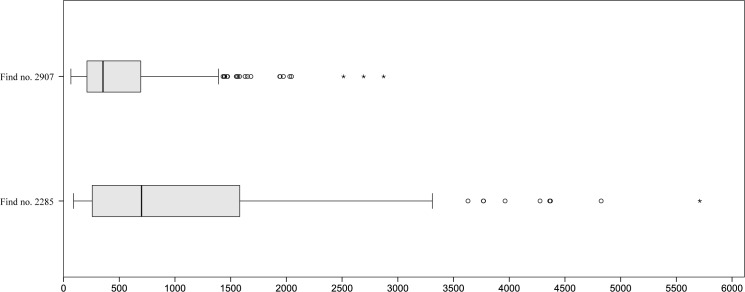
Grain size measurements of both finds. Symbols: °: extremal values, *: outliers. For raw data, see S1 and S3 Tables.

Find no. 2907, in contrast, shows a more homogeneous composition with a large proportion of flour/dunst grain sizes, therefore representing a more refined product (Fig 18). It must be emphasised that such interpretations will greatly profit from the availability of similar data for additional objects, and also from future experimental approaches [20].

#### Fermenting

Using only the very basic classification system according to Lannoy et al. [16], which only takes into account the overall height of a bread find, both bread-like objects from Zürich Parkhaus Opéra have to be classified as clearly unleavened, and therefore as flat breads (*galette*).

**Fig 19:**
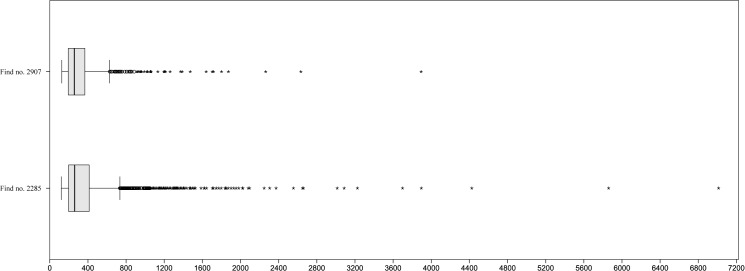
Cavity measurements of both finds. Symbols: °: extremal values, *: outliers. For raw data, see S2 and S4 Tables.

Current results from cavity/pore measurements provide additional quantitative evidence that can be used in confirming the validity of this classification: despite the differences in outlying values, general population parameters are nearly identical in both finds (Fig 19). In total, less than 5% of the cavities exceeded 1 mm in diameter. Although direct comparison to modern raised bread must be regarded with caution (also, but not only, because of the effects of charring), differences in magnitude are evident: image analysis values display percentages of larger (> 1mm) pores typically as high as 40% to 70% [119].

Taking into account the overall density of the finds with the naked eye and the lack of differentiated crust and crumb structure, the low height of the two finds and the aforementioned data, it can safely be assumed that neither of the two bread-like objects seem to have undergone any sufficient fermentation (and resulting gas development).

#### Shaping

The ring shape of find no. 2285 may be regarded as an indication of this bread-like object’s solid state prior to charring. However, this solid state may have been caused either by cooking/baking, or just by drying (see below). The function and purpose of the hole in this find remain uncertain for now. Although the phenomenon of punched bakery products is not new to archaeology or ethnography [32, 120–122], material that can be directly compared to find no. 2285 is not available.

#### Baking

Neither of the finds showed intact starch granules under the SEM. The absence of starch granules in processed cereal products is usually a consequence of full gelatinisation of the starch: In contrast to the reversible swelling of starch grains by soaking in water, gelatinisation requires elevated temperatures (the lower gelatinisation range of most starches lies between 55 and 75°C) [27, 123]. The presence of intact starch granules in charred material indicates that no soaking and subsequent cooking/baking processes occurred prior to charring [124]. In contrast, intact starch granules would be expected in any uncooked/unbaked dough, or in soaked flour/grain-paste [22]. The fully gelatinised starch in the analysed finds therefore indeed indicates dough preparation (soaking) and baking prior to charring.

The possible causes of charring also require elaboration, taking into account the two scenarios of “baking accident” vs. “catastrophic fire”. A fire event destroying certain houses located in the central part of the dwelling is indeed documented by a charcoal accumulation across more than 65 m² in Layer 13 (see Fig 2). Both finds, however, were discovered outside the area in question [81, 125], rendering the scenario of baking accidents for both objects more plausible.

## Conclusions

The two bread-like objects from the Neolithic site of Zürich Parkhaus Opéra are unleavened and baked cereal products with heterogeneous composition (grain chunks and ground flour). The ring-shaped find no. 2285 has a basis of a less refined, rougher milling product. In the tradition of other such bread finds, the two bread-like objects would be classified as flat breads [16, 21]. Find no. 2285 bears evidence of barley, and find no. 2907 of a mixture of wheat and barley as raw materials, documenting that flat breads can indeed be composed of other cereals than barley, and of mixtures of more than one cereal. The celery fruit in find no. 2907 seems to be the first evidence for the use of bread condiments in the European Neolithic. Given the concentration of grinding stones and bran fragments in the southern part of the settlement, the area where both bread-like objects were excavated, their place of discovery may very well be their place of production.

We suggest establishing a standardised and structured laboratory protocol for analysing archaeological “bread” remains in order to allow for a general comparability of data from such finds [14, 20, 45]. The current paper proposes a general framework for such an approach. Ann-Marie Hansson emphasised very fittingly, “(…) the necessity of looking upon our study material with an open mind and of not clouding our understanding of these old loaves by present-day bread concepts” [2].

## Supporting information

S1 Table Particle measurements of find no. 2285. Raw data of the histogram in Figs 13 and 17.
(XLSX)

S2 Table Cavity measurements of find no. 2285. Raw data of the histogram in Figs 14 and 18.
(XLSX)

S3 Table Particle measurements of find no. 2907. Raw data of the histogram in Figs 15 and 17.
(XLSX)

S4 Table Cavity measurements of find no. 2907. Raw data of the histogram in Figs 16 and 18.
(XLSX)
